# Pennsylvania Training School for Feeble-minded Children

**Published:** 1860-07

**Authors:** 


PENNSYLVANIA TRAINING SCHOOL FOR FEEBLE-MINDED CHILDREN.—This Institution, now in the eighth year of its existence, is situated near Media, in Delaware County, close by the new West Chester Railroad, and is under the superintendence of Dr. Joseph Parrish, who has made this class of unfortunates an object of special study. The exhibition which he gave of some of his pupils a year since, before the Pennsylvania State Medical Society, at its meeting in this city, was one of deep interest, and well calculated to excite the sympathy and enlist the good feeling of every one present on the occasion. The new edifice recently erected for the accommodation of the children, is said to be highly commodious, with every possible facility and comfort that such an establishment ought to possess. The grounds are large, and afford ample opportunity for horticultural and agricultural employment. Provision has also been made for regular, systematic gymnastic exercises. Last winter the Legislature made a handsome appropriation for the benefit of the Institution, which may now be regarded as reposing upon a permanent basis. If our space permitted, we should be strongly disposed to publish, entire, the admirable report of Dr. Parrish, a document which does infinite credit alike to his heart and his understanding. We trust it may be extensively circulated among all classes of people.

